# Incidence of Non-thrombotic Diagnoses Following Venous Duplex Ultrasound at a Community Emergency Department

**DOI:** 10.7759/cureus.16911

**Published:** 2021-08-05

**Authors:** Bailey Hasenbalg, Anthony Santarelli, Christopher Lyon, Shane Sergent, Heesun Choi, John Ashurst

**Affiliations:** 1 Emergency Medicine, Kingman Regional Medical Center, Kingman, USA; 2 Emergency Medicine, Duke Lifepoint Memorial Medical Center, Johnstown, USA; 3 Emergency Medicine, Michigan State, East Lansing, USA

**Keywords:** deep vein thrombosis (dvt), venous duplex ultrasound, non-thrombotic, community emergency department, lower extremity, upper extremity

## Abstract

Background: When used as a diagnostic aid for diagnosing deep vein thrombosis (DVT), venous duplex ultrasound (US) may reveal non-thrombotic findings in those with acute extremity pain. The objective of this study was to determine the prevalence and predictors of non-thrombotic findings on venous duplex US at a community emergency department.

Methods: A retrospective chart review of all adult patients who presented to a community emergency department who underwent either an upper or lower extremity venous duplex US for the evaluation of DVT from June 1, 2019, to September 15, 2020. All US studies were completed by certified sonographers and interpreted by board-certified radiologists. Two trained research assistants manually abstracted patient demographics and US findings. Data were analyzed using the chi-square statistic for categorical variables and the student's independent t-test for continuous variables. Multivariate binomial regression was used to identify independent predictors of non-thrombotic results on venous duplex US.

Results: A total of 1,448 venous duplex US were obtained during the study period with 126 DVTs being diagnosed. A total of 1071 US had no acute abnormality and 252 had non-thrombotic findings. All non-thrombotic findings were found in the lower extremity. Of those with non-thrombotic findings, the most common diagnoses included edema (34.9%, 88/252), Baker’s cyst (22.6%, 57/252), and an unspecified fluid collection (16.3%, 41/252). Patients with non-thrombotic findings were more likely to have a history of atrial fibrillation (p=0.001) or hypertension (p=0.001), be older than the age of 70 (p=0.042), or have a history of using illicit drugs (p=0.003). Females were less likely to have non-thrombotic findings.

Conclusion: In this single-site study, non-thrombotic findings were present in 23.5% of all venous duplex US completed at a community emergency department. These findings are more common in the elderly, those with cardiovascular disorders, and those who have used illicit drugs.

## Introduction

Concern for deep venous thrombosis (DVT) is a common reason patients present to the emergency department [1]. Following the presentation, patients are screened with venous duplex ultrasound (US) to detect either the presence or non-presence of a DVT [1]. Although thrombosis is one of the most worrisome diagnoses, emergency medicine providers should also include non-thrombotic findings in the differential diagnosis that include edema, hematoma, baker’s cyst, and venous insufficiency [1].

Venous duplex US has become the imaging modality of choice for the diagnosis of DVT due to its sensitivity and specificity [1]. Although used to diagnose DVTs, US often also identifies non-thrombotic pathology in the upper and lower extremities [2,3]. Data from large tertiary care facilities have shown that a large number of US used to detect DVTs diagnose non-thrombotic pathology as a cause for a patient’s symptoms [2,3]. Although data on the non-thrombotic findings on venous duplex US exist for tertiary care facilities, little to no data has been reported on the prevalence of and predictors for non-thrombotic findings venous duplex US at a community emergency department. Prior data on non-thrombotic findings also did not include findings from the upper extremities [2,3]. The authors sought to determine the prevalence and predictors of non-thrombotic findings following an initial venous duplex US of the upper and lower extremities at a community emergency department.

## Materials and methods

Study location

Kingman Regional Medical Center is a 235-bed rural community hospital in northwestern Arizona with an annual emergency department census of approximately 55,000 patient visits.

Protocol

Following Institutional Review Board approval, a retrospective chart review was conducted from June 1, 2019, through September 15, 2020, for adult patients (age ≥ 18) who had an upper or lower extremity venous duplex US completed as part of their emergency department care. Patients who underwent portable (point-of-care) ultrasonography, adults with associated pulmonary embolism, and patients with a DVT were excluded from the analysis. If a patient underwent multiple US during the study period, all were included in the analysis unless it met one of the exclusion criteria. All venous duplex USs were completed by certified sonographers following institutional protocols and interpreted by board-certified radiologists. With adherence to a quality-controlled protocol and structured abstraction tool, two trained research assistants manually collected patient demographics and venous duplex results. For those with missing data, the primary investigator reviewed each case and removed the case if not able to locate the missing data point. Research assistants were blinded to the primary outcome of the study until all data points had been collected. Abstractor monitoring and verification of the independent variables were completed by the primary investigator.

Data analysis

Statistical analysis was conducted using IBM SPSS statistics version 27 (IBM Corp., Armonk, NY, USA). Continuous data are presented as the mean and 95% confidence interval and compared using an independent samples t-test. Categorical data are presented as frequencies with percentages of the sample and were analyzed using the chi-squared statistic. A stepwise multivariate binomial logistic regression was used to determine the most influential factors that predict the presence of a non-thrombotic finding. A-priori variables selected for regression included age and gender, while post-hoc significant variables were upon intergroup differences.

## Results

A total of 1,448 extremity venous duplex ultrasounds were reviewed and 137 were of the upper extremity and 1,311 of the lower extremity. A total of 58.5% (847/1,448) of all ultrasounds were obtained in females and 41.5% (601/1,448) in males. There were 118 DVTs located in the lower extremity (93.6%) and eight (6.4%) in the upper extremity.

The majority (73.9%, 1,070/1,448) of venous duplex US obtained during the study period for extremity pain showed no acute abnormality. Non-thrombotic findings were found in a total of 17.5% (252/1,448) of the patients who underwent venous duplex US. All non-thrombotic findings occurred in the lower extremity (19.2%, 252/1,311). The average age of patients with non-thrombotic findings was 64.6 years. The most common non-thrombotic findings in the cohort were edema (34.9%, 88/252), Baker’s cyst (22.6%, 57/252), and an unspecified fluid collection (16.3%, 41/252) (Table 1). Females were more likely to have a Baker's cyst as compared to their male counterparts (29.23% vs 15.32%; p=0.017).

**Table 1:** Non-thrombotic findings in the lower extremity following venous duplex ultrasound examination

	Total ( N=252)	Female (N =128)	Male (N =124)	
Finding	N (% of Total)	N (% of Female)	N (% of Male)	P-value
Superficial Thrombosis	27 (10.71%)	13 (10%)	14 (11.29%)	0.79
Edema	88 (34.92%)	40 (30.77%)	48 (38.71%)	0.32
Unspecified Fluid Collection	41 (16.27%)	20 (15.38%)	21 (16.94%)	0.80
Baker's Cyst	57 (22.62%)	38 (29.23%)	19 (15.32%)	0.017
Knee Effusion	10 (3.97%)	6 (4.62%)	4 (3.23%)	0.57
Post-Thrombotic Finding	9 (3.57%)	5 (3.85%)	4 (3.23%)	0.79
Tissue Mass	3 (1.19%)	1 (0.77%)	2 (1.61%)	0.56
Arterial Finding	5 (1.98%)	2 (1.54%)	3 (2.42%)	0.66
Lymphedema	12 (4.76%)	3 (2.31%)	9 (7.26%)	0.073

Patients greater than 70 years of age were more likely to have non-thrombotic findings (p=0.042) and patients younger than 40 years of age were less likely to have non-thrombotic findings (p=0.003) (Table 2). Non-thrombotic findings were also more common among patients with a history of hypertension (52.6% vs 64%; p=0.001) or atrial fibrillation (8.4% vs 15%; p=0.001) (Table 2). Females were less likely to have non-thrombotic findings compared to their male counterparts (p=0.018). Patients using illicit drugs were also more likely to have non-thrombotic findings (5.9% vs 11.1%; p=0.003) than those not using illicit drugs. There was no difference in the proportion of non-thrombotic findings in cancer patients, those with a prior DVT, those on hormone replacement therapy, or those taking birth control.

**Table 2:** Demographic and social predictors for non-thrombotic findings in patients evaluated with venous duplex ultrasound DVT - deep vein thrombosis

	All patients without DVT	No finding	Non-thrombotic finding	P-value
Age				
Aged less than 40 (y)	202	186 (15.6%)	16 (6.3%)	0.003
Aged 41-70 (y)	758	624 (52.2%)	134 (53.2%)	0.82
Aged over 70 (y)	487	385 (32.2%)	102 (40.5%)	0.042
Medical History				
Hypertension	791	630 (52.6%)	161 (64.0%)	0.001
A-fib	138	100 (8.4%)	38 (15.0%)	0.001
Cancer	158	129 (10.7%)	29 (11.9%)	0.60
Prior DVT	255	207 (17.2%)	48 (19.4%)	0.42
Social History				
Smoking	382	308 (25.8%)	74 (29.2%)	0.26
Alcohol	418	341 (28.5%)	77 (30.4%)	0.55
Drug Use	98	70 (5.9%)	28 (11.1%)	0.003
Medication				
Birth Control	24	23 (1.9%)	1 (0.4%)	0.08
Hormone Therapy	55	47 (3.9%)	8 (3.2%)	0.36

The stepwise logistic regression conducted to evaluate whether the patient demographics and social histories predict the detection of a non-thrombotic finding revealed a three-step model with age (OR=1.02; p<0.001), drug use (OR=2.32; p<0.001), and atrial fibrillation (OR=1.57; p=0.034) entered as sequential independent predictors of non-thrombotic finding (Table 3).

**Table 3:** Multiple logistic analysis for the predictors of non-thrombotic and no acute findings in patients evaluated with venous duplex US for extremity pain

Predictor	Odds ratio	95% confidence interval	P-value
Age (per year)	1.02	1.01-1.03	<0.001
Drug use	2.32	1.45-3.72	<0.001
Atrial fibrillation	1.57	1.04-2.38	0.034

## Discussion

Of the venous duplex USs reviewed, there were non-thrombotic findings in 17.5% of all lower extremity studies with edema, Baker’s cyst, and unspecified fluid collection being the most common findings reported. In previous studies analyzing the prevalence of non-thrombotic findings in venous duplex US of the lower extremity, the prevalence of non-thrombotic findings ranged between 11% and 65.7% [2-5]. The difference in prevalence between the current study and the reported literature could be related to the number of US obtained for lower extremity pain and the lack of an institutional protocol to rule out DVT. Another difference that may have led to the difference in prevalence between the current study and the reported literature is the technique used to acquire US images (vascular technicians and subspecialized radiologists) and interpretation of US findings (vascular surgeons or subspecialized radiologists) [4,5].

The most common findings in the previous studies assessing non-thrombotic findings in the lower extremity were venous valvular incompetence, cyst/mass, edema, lymphadenopathy, and superficial thrombosis [2-5]. The results of the current study replicate previous reports with the most common findings being edema, cyst, fluid collection, and superficial thrombosis [2-5]. The convergence of the results from data collected at academic medical centers and now community hospitals suggests that non-thrombotic findings are relatively homogenous between patients in rural and urban settings [2-5].

Given the prevalence and types of non-thrombotic findings in those who underwent venous duplex US, a focused evaluation of the venous system could be insufficient in select patient populations. The current results identified important differences in demographic factors between those with and without non-thrombotic findings on venous duplex US who underwent venous duplex US in the ED. Younger patients were unlikely to present with a non-thrombotic finding in the extremities but older patients were more likely to present with non-thrombotic findings. These results are consistent with prior literature that showed that each one-year increment in age was associated with an increased likelihood of finding a non-thrombotic cause on the whole-leg US [4]. The current study also found that both atrial fibrillation and drug use were significant predictors for a non-thrombotic finding in those undergoing venous duplex US at a community emergency department.

Limitations

An important limitation to this study is that it was a retrospective chart review of ED patients conducted at a single rural community hospital in a relatively homogenous population. This may limit the generalizability of the results to other rural populations and tests obtained by other providers. This study also did not assess the clinical outcomes or significance of those with non-thrombotic findings. Board-certified radiologists reviewed all venous duplex USs and may not have included all non-thrombotic findings given each test was ordered to assess the venous vascularity of the extremity.

## Conclusions

The prevalence of non-thrombotic findings for adults undergoing venous duplex US at a single community ED was 23.5%. Older patients, those with a history of drug use or atrial fibrillation, are most at risk for the development of a non-thrombotic finding on venous duplex US. Patients meeting these criteria with a chief complaint of lower extremity pain presenting to a community emergency department should be administered venous duplex US to rule out DVT and a focused diagnostic US to rule out non-thrombotic diagnoses.
